# Small‐scale and multi‐species approaches for assessing litter decomposition and soil dynamics in high‐diversity forests

**DOI:** 10.1002/aps3.1241

**Published:** 2019-04-19

**Authors:** Francesco Martini, Shang‐Wen Xia, Xiaodong Yang, Uromi Manage Goodale

**Affiliations:** ^1^ Guangxi Key Laboratory of Forest Ecology and Conservation College of Forestry Guangxi University Daxuedonglu 100 Nanning Guangxi 530004 People's Republic of China; ^2^ State Key Laboratory of Conservation and Utilization of Subtropical Agro‐Bioresources College of Forestry Guangxi University Daxuedonglu 100 Nanning Guangxi 530004 People's Republic of China; ^3^ Ailaoshan Station for Subtropical Forest Ecosystem Studies Chinese Ecosystem Research Net Jingdong Yunnan 676200 People's Republic of China; ^4^ Key Laboratory of Tropical Forest Ecology Xishuangbanna Tropical Botanical Garden Chinese Academy of Sciences Xishuangbanna 666303 People's Republic of China

**Keywords:** home‐field advantage, litter decomposition, microbial nutrients, soil nutrients, subtropical forest, tree species identity

## Abstract

**Premise of the Study:**

The relationship between tree species abundance and diversity and soil chemistry has been studied in several ecosystems and at different spatial scales. However, species‐specific assessments have mainly been conducted in temperate ecosystems and in monospecific settings, calling for studies from diverse, mixed forests from different ecosystems.

**Methods:**

In a subtropical forest in southern China, under four dominant tree canopy species (*Lithocarpus chintungensis*,* Castanopsis wattii*,* Schima noronhae*, and *Manglietia insignis*), we assessed species’ effect on inter‐ and intraspecific percentages of litter mass loss, and the effect of species on soil nutrients and soil microbial biomass.

**Results:**

Our results show significant differences in litter decomposition for all four species; however, the percentage of litter mass loss was stable under different species. Microbial biomass and soil nutrients presented strong differences under different tree species. Species‐specific differences in soil characteristics were seen for carbon‐nitrogen‐phosphorus relationships. Surprisingly, the correlations between carbon and phosphorus and between nitrogen and phosphorus showed opposite slopes in soils collected under different tree species.

**Discussion:**

Our results provide insights into the importance of tree species identity in providing variety to ecosystem processes occurring on the forest floor. We recommend this methodological approach—combining analysis of litter decomposition, soil nutrient concentrations, and microbial biomass—when dealing with species‐rich forests.

Litter decomposition is a key factor in maintaining ecosystem function, nutrient cycling, and carbon fluxes (Swift et al., 1979; Makkonen et al., 2012). These processes are influenced by abiotic and biotic factors, which differ in their relative importance at the global or local scale. Globally, climatic conditions play a major role, contributing for example to most of the variation in litter decomposition, microbial community composition, and soil characteristics (Aerts, 1997; Gholz et al., 2000). Plant litter quality and the decomposing organisms, both in terms of quality and abundance, also contribute to global variation in litter decomposition (Couteaux et al., 1995; Cornwell et al., 2008). However, approximately 30% of the variation in litter decomposition can be explained by other variables that may be acting at local scales (Austin and Vivanco, 2006; Hobbie et al., 2006; Ayres et al., 2009a).

One mechanism that has been proposed to describe part of this remaining variation is the home‐field advantage (HFA) hypothesis, which predicts that litter will decompose faster in its “home” habitat (i.e., around the plant species from which it originates) rather than away from it, likely because of adapted decomposer communities (Gholz et al., 2000; Ayres et al., 2009a; Austin et al., 2014). This hypothesis has been tested in different habitats, ecosystems, and in laboratory experiments, providing contrasting results: it has been alternately confirmed (Ayres et al., 2009b; Milcu and Manning, 2011; Veen et al., 2015), dismissed (Gießelmann et al., 2011; St. John et al., 2011), and shown mixed results (Chomel et al., 2015; Jewell et al., 2015; Sun and Zhao, 2016). Most of the literature on the HFA hypothesis, and on litter decomposition more broadly, has assessed the validity of this hypothesis with reciprocal transplant experiments between two or more ecosystems (forest vs. grassland) or different forest types, both in the field (Horodecki and Jagodzinski, 2017; Parker et al., 2018) and in common gardens (Hobbie et al., 2006). However, almost all of these studies have been conducted in the Americas or Europe in temperate or tropical systems, with an underrepresentation of the Asian continent and of subtropical forests (see fig. 1 in a recent review from Veen et al., 2015). Furthermore, previous studies that assessed species‐specific litter decomposition in natural environments were often developed in pure or almost pure stands of the selected species (Ayres et al., 2009b; Milcu and Manning, 2011; Horodecki and Jagodzinski, 2017). Of those studies that assessed litter decomposition of a single litter species, litterbags were placed in different forest mixtures (Barlow et al., 2007; Trogisch et al., 2016). These investigations provide useful information about how the litter of different species decomposes and the influence of different habitats on decomposition and the release of nutrients. Nonetheless, small‐scale assessments in primary mixed forests are lacking, with very few studies conducted in subtropical species‐rich forest and assessing decomposition under individual trees of selected species. For example, Vivanco and Austin (2008) assessed the species‐specific effect of different tree species on litter decomposition in a *Nothofagus* Blume mixed forest in Patagonia. They found that litter was decomposing faster in its home site, but the biogeochemical characteristics (e.g., soil pH, carbon [C], nitrogen [N]) of each microsite where litterbags were placed did not differ significantly. However, the forest had low diversity and was found to be primarily composed of *Nothofagus* species (rather than being a mixed‐species forest); therefore, this study does not provide information on species‐rich forests.

**Figure 1:**
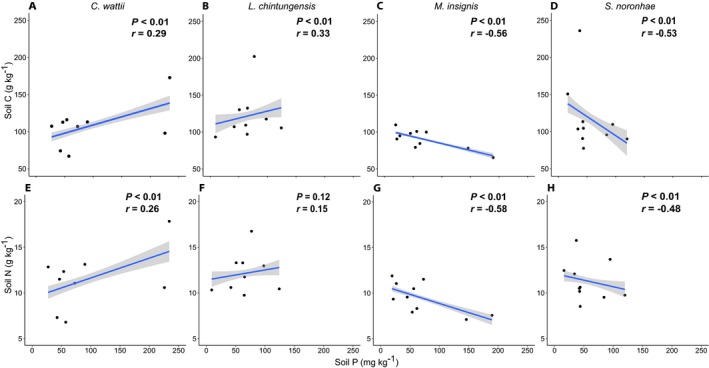
Spearman's rank correlations between soil carbon, nitrogen, and phosphorus under different tree species: *Castanopsis wattii* (A, E), *Lithocarpus chintungensis* (B, F), *Manglietia insignis* (C, G), and *Schima noronhae* (D, H). *P* values and Spearman's rank correlation coefficients are shown.

Similar to litter decomposition, soil characteristics are also known to present high local heterogeneity, including soil nutrients (Xia et al., 2016) and microbial biomass (Scheu et al., 2003). In both cases, globally constant linear relationships exist among C, N, and phosphorus (P). This was first found for marine organisms, when Redfield (1958) described a constant stoichiometric relationship in planktonic biomass and marine water, and has subsequently been investigated in terrestrial ecosystems (Reich and Oleksyn, 2004; Cleveland and Liptzin, 2007). Most of these studies were conducted at larger spatial scales, while less attention has been paid to fine‐scale processes. One example is Vesterdal et al. (2008), who assessed the composition in C and N on the forest floor, under six tree species, using a common garden experiment in monoculture stands. The rarity of studies conducted in species‐rich natural forests precludes inference of these processes in forest dynamics. In summary, we have a limited understanding of the local processes affecting decomposition and soil characteristics and the interactions among them, especially in diverse ecosystems (Table 1).

**Table 1:** Summary of selected studies on litter decomposition (and the home‐field advantage [HFA] hypothesis), their approaches, and outcomes.^a^^,^ b

Reference	Study site	Study setting	Litter species	Litter location level	Litter decomposition (HFA)^c^	Soil nutrients	Microbial biomass (N, C, P)
This study	**Subtropical forest** (China)	**Natural forest**	4	**Species** (individual trees)	X (Negative)	X	X
Aponte et al., 2012	Mediterranean oak forest (Spain)	**Natural forest**	2	**Species**/forest site			
Ayres et al., 2009a	Review	Several			X (Positive)		
Barlow et al., 2007	Amazonia (Brazil)	Primary, secondary, plantation forests	4	Forest type	X (Negative)		
Chomel et al., 2015	Boreal region (Canada)	Forest plantations	2	Plantation type	X (Mixed)		
Gießelmann et al., 2011	Atlantic rainforest (Brazil)	Secondary forest	Mixed litter	Successional stage	X (Negative)		
Hobbie et al., 2006	Temperate region (Poland)	Pure stands	14	Stand type	X (Negative)		
Horodecki and Jagodzinski, 2017	External spoil heap (Poland)	Pure stands	6	Stand type	X (Positive)		
Jewell et al., 2015	Boreal region (Canada)	Forest plantations	4	Plantation type	X (Mixed)		
Liu et al., 2000	**Subtropical forest** (China)	**Natural forest**	3	Forest floor			
Liu et al., 2005	Tropical/**subtropical forest**	**Natural forest**	2 + mixed litter	Forest type			
McGuire et al., 2010	Tropical forest (Guyana)	Mono‐dominant/mixed tropical rainforest	1 + mixed litter	Forest type	X (Negative)	X	
St. John et al., 2011	Patches of kanuka (New Zealand)	Mono‐dominant patches	1 + mixed grasses	Forest/grassland	X (Negative)		
Sun and Zhao, 2016	Urban forest park (China)	Pure stands	2	Stand type	X (Positive)		
Trogisch et al., 2016	**Subtropical forest** (China)	Plots with different diversity and age	26	Forest type (plots)			
Veen et al., 2015	Review	Several			X (Mixed)		
Vivanco and Austin, 2008	Temperate region (Argentina)	**Natural forest**	3	**Species** (“triangles”)	X (Positive)	X	X

a All studies are referenced in the manuscript. Although these studies do not present a complete review of the existing literature, they highlight the following main knowledge gaps addressed in our study: (1) subtropical and natural forests are underrepresented; (2) litter decomposition is often assessed at the species level, but rarely under a species’ own canopy (same species) compared to decomposition under different species' canopies (HFA hypothesis); and (3) litter decomposition is rarely assessed together with soil characteristics and microbial biomass (but microbial community composition has been studied in McGuire et al. 2010 and St. John et al. 2011).

b Bold text represents aspects of the studies that fill one or more of the above‐mentioned knowledge gaps (marked with an ‘X’).

c Results that did not meet the HFA hypothesis are marked as “Negative,” studies that supported the HFA hypothesis are marked as “Positive,” and mixed results are identified as “Mixed.”

The aim of this study was to investigate the effect of tree species on forest floor processes, including litter decomposition, soil nutrients, and microbial biomass—here measured as C and N—using a fine‐scale methodological approach. One subtropical forest in Ailao Mountain, southern China, was chosen as an ideal site due to its high species richness—up to 94 tree species in 0.4 ha (Young and Herwitz, 1995). We selected four dominant tree canopy species: *Lithocarpus chintungensis* Hsu & Qian, *Castanopsis wattii* A. Camus, *Schima noronhae* Reinw., and *Manglietia insignis* Blume. Under each tree, we measured litter mass and assessed the species’ effect on inter‐ and intraspecific percentage of litter mass loss to test the HFA hypothesis. We also assessed differences in soil nutrients, and in soil microbial biomass C and N, using measures from under individual trees. The litter decomposition differences among the four selected species were tested using a reciprocal transplant experiment and the litterbag method (Swift et al., 1979; Horodecki and Jagodzinski, 2017). Specifically, we tested three hypotheses: (1) litter decomposition is faster under a species’ own canopy (same species) compared to decomposition under different species’ canopies (HFA hypothesis), (2) soil characteristics show significant variation under the canopy of different tree species, and (3) soil microbial biomass is significantly affected by the canopy species’ tree identity.

## METHODS

### Study site and tree selection

This study was conducted on Ailao Mountain, Xujiaba region (24°32′22″N, 101°01′36″E), in Yunnan Province, southwestern China (Appendix 1), at approximately 2540 m elevation. The climate is subtropical monsoon, with an annual precipitation of about 1900 mm and 85% of the rain falling in the rainy season between May and October. The mean annual temperature is 11.3°C, and the mean monthly temperature is 16.4°C and 5.4°C for the warmest (July) and the coldest (January) months, respectively. The frost‐free period is about 200 days per year. Soil type is typically yellow‐brown earth, with a loamy soil texture, and pH ranges from 4.4–4.9 (Qiu et al., 1998). A litter layer 3–7‐cm thick usually covers the ground. The forest type is mid‐montane humid evergreen broadleaved forest. Dominant tree species in the canopy are *L. xylocarpus* Markg., *L. chintungensis*,* C. wattii*,* Machilus viridis* Hand.‐Mazz., *Manglietia insignis*, and *S. noronhae* (Liu et al., 2002).

Four canopy‐dominant, evergreen tree species (*C. wattii*,* L. chintungensis*,* M. insignis*, and *S. noronhae*) were chosen for this study (for species information, see Appendix 2). In August 2015, 10 adult trees of each species were randomly selected and marked, resulting in a total of 40 trees. Later in the experiment, we found that one *L. chintungensis* tree had been misidentified as *C. wattii*. After this correction, the number of individuals per study species was 10 each for *M. insignis* and *S. noronhae*, nine for *C. wattii*, and 11 for *L. chintungensis*.

### Litter decomposition experiment

In January 2015, fallen leaves of the four selected species were collected from the forest floor and air‐dried for one month. In August 2015, 5 g of air‐dried litter of each species was placed into litterbags with 2‐mm mesh size. Three litterbags per species were laid under each of the selected trees, for a total of 480 litterbags (4 species **×** 3 litterbags **×** 40 trees). The initial mass of litter for each species was calculated using three randomly chosen bags for each species, which were oven‐dried for 24 h at 80°C, and then weighed (Aponte et al., 2012). These mass data were later used in the analysis as initial litter mass per species. In August 2017, all litterbags were retrieved, and non‐litter material including soil and insects was carefully removed. The remaining litter was then oven‐dried at 80°C for 24 h and weighed again with a precision of 0.0001 g (AL104 Analytical Balance; Mettler‐Toledo Instruments, Shanghai, China).

### Soil characteristics and microbial biomass C and N

For each individual tree, three soil cores were collected in August 2015 with a 4‐cm‐diameter and 10‐cm‐depth soil probe, within a distance of 1 m from the individual trunk. The three soil cores were then mixed in the field before being transferred to the Xishuangbanna Tropical Botanical Garden Central Laboratory for analysis. All samples were cleaned by removing stones, leaves, and other debris before analysis. Each sample was divided into 25 g for the soil microbial biomass assessment, and the rest was air‐dried and stored for other soil nutrient measurements, including total C, total N, available P, and available potassium (K).

Soil pH was measured with a pH meter (PHS‐3C; Shanghai Precision Scientific Instrument Co. Ltd., Shanghai, China) in water, using 2.5 g of deionized water per 1 g of soil. Total C and N were measured with a carbon–nitrogen analyzer (Vario MAX CN; Elementar Analysensysteme, Langenselbold, Germany). Available P and K were extracted with Mehlich III solution (John et al., 2007; Tran and Ziadi, 2007), and a spectrophotometer (T723; Shanghai Spectrum Instruments Co. Ltd., Shanghai, China) was used to measure the available P concentration; for available K, we used an inductively coupled plasma atomic‐emission spectrometer (IRIS Advantage‐ER; Thermo Jarrell Ash Corporation, Waltham, Massachusetts, USA).

Microbial biomass C and N were determined using the chloroform fumigation–direct extraction method (Brookes et al., 1985).

### Statistical analysis

We calculated litter decomposition as percentage of dry litter mass loss from initial time to the collection time, two years later:

$$\%massloss\;=\;100\;-\;(\frac{m}{\mathrm{im}}*100)$$

where *m* is the remaining dry litter mass at time *t* and *im* is the initial dry litter mass. To test the effect of tree species identity on litter decomposition (hypothesis 1), we conducted a species‐by‐species assessment of litter mass loss for each species’ litter placed under its own canopy against mass loss of the other species’ litter under the same canopy tree species, using a one‐way ANOVA followed by multiple comparisons using the *multcomp* R package (three comparisons; Hothorn et al., 2008). If needed, arcsine transformations were applied to correct non‐normal data. We further tested if there were any significant differences between litter decomposition in the home habitat compared to litter in the away habitat using a *t*‐test. For non‐normal data (i.e., the *L. chintungensis* and *M. insignis* litter data), we used the nonparametric Mann–Whitney *U* test. Here, for the away habitat, we pooled together the litter mass loss of the litterbags placed under the other three tree species and obtained the mean to test against the decomposition of the litter in the home habitat. In order to test our second and third hypotheses, namely that the concentrations and relationships of soil characteristics (soil pH, C, N, P, and K) and microbial biomass (C and N) differ under different tree species, we first used Kruskal–Wallis nonparametric tests that are appropriate for data that do not meet the assumptions of normality and/or homogeneity of variance. Dunn post‐hoc tests for multiple comparisons were then applied at a significance level of *P* < 0.05. Spearman's rank correlation from the *agricolae* package (Mendiburu, 2017) was used to assess the relationship between soil characteristics and microbial biomass under different tree species. All statistical analyses were carried out using R (version 3.4.4; R Core Team, 2018), and all figures displaying statistical data (including appendix figures) were produced through the R package *ggplot2* (Wickham, 2009).

## RESULTS

### Litter decomposition

Percentage of litter mass loss was significantly different among tree species (Kruskal–Wallis: *χ* ^2^ = 132.62, *df* = 3, *P* < 0.01). Litter of *C. wattii* decomposed the most (87.48% ± 0.79 mass loss), whereas litter of *L. chintungensis* decomposed the least (71.14% ± 0.94; Appendix 3A). However, there were no significant differences in percentage of litter mass loss under different tree species when litter from all four litter species were pooled together (Kruskal–Wallis: *χ* ^2^ = 5.3005, *df* = 3, *P* = 0.15; Appendix 3B). Litter mass loss was not higher in the home habitat compared to the away habitat. Among litter species, only litter decomposition from *M. insignis* was significantly different under different tree species (*F*_3,115_ = 3.583, *P* = 0.016), with litter under the home habitat decomposing significantly more than under *L. chintungensis* tree species, but not more than under *C. wattii* and *S. noronhae* (Appendix 4C). Litter of the other three species did not show differences in decomposition when placed under any other tree species (Appendix 4A, B, and D). These results were confirmed by the *t*‐test and Mann–Whitney *U* test, with all the litter species not decomposing significantly faster in the home habitat compared to away from it (Appendix 4E–H). When isolating litter mass loss under each individual tree species, the overall results were confirmed, with significant species‐specific differences in litter mass loss. In all cases, *C. wattii* litter exhibited the highest mass loss, regardless of the tree species it was deposited under, whereas litter from *L. chintungensis* showed the lowest mass loss in all cases, except under *S. noronhae*. A summary of the litter decomposition values for all species can be found in Table 2 and Appendix S1.

**Table 2:** Summary of litter decomposition under different tree species, expressed as percentage of litter mass loss.^a^

Litterbag location	*Castanopsis wattii*	*Lithocarpus chintungensis*	*Manglietia insignis*	*Schima noronhae*
Total	87.48 ± 0.79 (119)	71.14 ± 0.94 (118)	81.75 ± 1 (119)	74.75 ± 1.03 (118)
Under *C. wattii*	**88.62** a ^**,**^ a ± **1.54 (26)**	74.2^a^ ^,b^ ± 2.19 (26)	84.44^a^^,^ a ± 1.92 (27)	73.26^a^ ^,b^ ± 2.05 (27)
Under *L. chintungensis*	86.40^a^ ^,b^ ± 1.71 (33)	**70.61** a ^**,**^ a ± **1.41 (33)**	77.36^a^ ^,b^ ± 1.76 (32)	75.61^a^^,^ a ± 2.1 (33)
Under *M. insignis*	88.72^a^ ^,b^ ± 1.17 (30)	71.03^a^ ^,b^ ± 2.16 (29)	**85.15** a ^**,**^ a ± **1.64 (30)**	75.54^a^^,^ a ± 2.13 (29)
Under *S. noronhae*	86.44^a^ ^,b^ ± 1.76 (30)	69.19^a^^,^ a ± 1.82 (30)	80.62^a^^,^ a ± 2.35 (30)	**74.35** a ^**,**^ a ± **2.02 (29)**

a Numbers in parentheses represent the sample size (number of litterbags). Different letters represent significant differences resulting from the multiple comparisons following the one‐way ANOVA; the first letter represents differences between the same litter species under different tree species (columns), and the second letter represents differences between different litter species under the same tree species (rows). Comparisons are only made between the reference species (same species, same tree)—presented in boldface text—and each of the other species, but not between the other three species.

### Soil characteristics

Soil characteristics showed significant variation under different tree species (Appendix 5). Soil pH was significantly higher under *M. insignis* (4.42 ± 0.03) compared to the other species (Kruskal–Wallis: *χ* ^2^ = 51.152, *df* = 3, *P* < 0.01). Soil total C differed significantly among samples collected under different tree species (Kruskal–Wallis: *χ* ^2^ =110.66, *df* = 3, *P* < 0.01), with the highest C found under *L. chintungensis* (122.26 ± 2.54 g·kg^−1^) and the lowest under *M. insignis* (90.1 ± 1.17 g·kg^−1^). Soil total N was different as well (Kruskal–Wallis: *χ* ^2^ = 87.916, *df* = 3, *P* < 0.01), with similar species differences found for soil C. Similarly, soil available P concentration (Kruskal–Wallis: *χ* ^2^ = 26.061, *df* = 3, *P* < 0.01) and soil available K (Kruskal–Wallis: *χ* ^2^ = 17.384, *df* = 3, *P* < 0.01) showed significant differences. Soil P showed the highest value in soil collected under *C. wattii* (93.69 ± 7.2 mg·kg^−1^) and the lowest value in soil collected around the trunks of *S. noronhae* (55.95 ± 2.84 mg·kg^−1^). For soil K, the highest amount was found under *M. insignis* (319 ± 5.81 mg·kg^−1^) and the lowest under *S. noronhae* (282.45 ± 7.15 mg·kg^−1^). A summary of the soil characteristics under each species is shown in Table 3.

**Table 3:** Soil nutrient concentration and microbial biomass carbon and nitrogen under four tree species in Ailao Mountain (values are means ± SE)

Soil/microbial variables	*Castanopsis wattii*	*Lithocarpus chintungensis*	*Manglietia insignis*	*Schima noronhae*
Soil pH	4.21 ± 0.03	4.20 ± 0.03	4.42 ± 0.03	4.09 ± 0.02
Soil total C (g·kg^−1^)	107.63 ± 2.79	122.26 ± 2.54	90.1 ± 1.17	117.33 ± 4.04
Soil total N (g·kg^−1^)	11.5 ± 0.3	12.2 ± 0.17	9.48 ± 0.1	11.31 ± 0.19
Soil‐available P (mg·kg^−1^)	93.69 ± 7.2	66.39 ± 2.97	68.83 ± 4.89	55.95 ± 2.84
Soil‐available K (mg·kg^−1^)	303.65 ± 3.78	293.5 ± 6.36	319 ± 5.81	282.45 ± 7.18
Microbial C (μg·g^−1^)	109.16 ± 2.7	125.8 ± 1.64	116.78 ± 2.87	114.09 ± 1.92
Microbial N (μg·g^−1^)	20.39 ± 0.33	24.03 ± 0.26	22.29 ± 0.4	20.89 ± 0.2

Correlation between soil C and N was positive under all species, ranging from 0.80 under *S. noronhae* to 0.93 under *M. insignis*. At the species level, strong differences in the correlations between soil C and P and between N and P were found (Fig. 1). The correlation between soil P and soil C was positive in *L. chintungensis* (*r* = 0.33, *P* < 0.01) and *C. wattii* (*r* = 0.29, *P* < 0.01), but negative in *M. insignis* (*r* = −0.56, *P* < 0.01) and *S. noronhae* (*r* = −0.53, *P* < 0.01). Similarly, the correlation between soil P and soil N was positive for *L. chintungensis* (*r* = 0.15, *P* = 0.12 [not significant]) and *C. wattii* (*r* = 0.26, *P* < 0.01), but negative in *M. insignis* (*r* = −0.58, *P* < 0.01) and *S. noronhae* (*r* = −0.48, *P* < 0.01).

### Microbial biomass C and N

Microbial biomass C (Kruskal–Wallis: *χ* ^2^ = 20.427, *df* = 3, *P* < 0.01) and N (Kruskal–Wallis: *χ* ^2^ = 95.295, *df* = 3, *P* < 0.01) showed significant differences under different tree species. In both cases, the microbial biomass was highest in *L. chintungensis* (125.8 ± 1.64 and 24.03 ± 0.26 μg·g^−1^ for microbial C and N, respectively) and lowest under *C. wattii* (109.16 ± 2.70 and 20.39 ± 0.33 μg·g^−1^ for microbial C and N, respectively; Appendix S2). The correlation between C and N was very strong when all samples were combined together (*r* = 0.75, *P* < 0.01), and the same positive slope was significant for all species. However, the Spearman's rank correlation coefficient ranged from 0.30 in soils under *L. chintungensis* to 0.95 in soils under *M. insignis*. Values of microbial biomass are presented in Table 3.

## DISCUSSION

In contrast to what was expected under the HFA hypothesis, litter did not decompose faster when placed under the “home” tree compared to when it was placed under different tree species, either when paired comparisons were done or when compared to the mean of all of the “away” species. Only for one species*, M. insignis*, did litter register a slightly higher decomposition in the home habitat; the 4.54% increase in decomposition shown for *M. insignis* is close to what was found in previous studies that confirmed the HFA hypothesis (Ayres et al., 2009b; Veen et al., 2015). Our overall results agree instead with studies that rejected the HFA hypothesis (Barlow et al., 2007; Gießelmann et al., 2011; St. John et al., 2011) or did not fully support it (Chomel et al., 2015; Jewell et al., 2015; Sun and Zhao, 2016). This could be explained by differences in litter quality, which may play a more relevant role on decomposition, as stated by Cornwell et al. (2008) in their global analysis on the main drivers of litter decomposition, and confirmed by other authors (Aponte et al., 2012; Makkonen et al., 2012). Data available from Liu et al. (2000, 2002) on the species studied here show that leaf nutrient concentrations are significantly different between the species. In Liu et al. (2000), it was proposed that decomposition is controlled by the initial concentration of N, P, and lignin. These findings have been confirmed by other authors (Hobbie et al., 2006; Berg, 2014).

The adaptation of decomposers to the local litter has been proposed by Ayres et al. (2009a) as the main mechanism explaining the HFA hypothesis. They hypothesized that, because of differences in the physical structure and chemical composition of litter, different soil biota are found under different plant species and these organisms are more specialized to decompose that specific litter. However, under natural conditions it is unlikely that litter found under a species has only originated from that tree's crown. This study was conducted in a diverse forest, where the litter under any given species may come from multiple species. Hence, it could be expected that the microbial community is diverse under any given tree crown. Indeed, our results suggest that the soil decomposer community is not adapted in a species‐specific manner at such a small spatial scale (i.e., at the individual tree species level), and that litter under a given tree species is diverse and thus cultivates a diverse decomposer community. Previous studies that assessed species‐specific litter decomposition in natural environments were often developed in pure or almost pure stands of the selected species (Ayres et al., 2009b; Milcu and Manning, 2011; Horodecki and Jagodzinski, 2017). In several other studies that assessed litter decomposition of a single tree species, litterbags were placed in different forest mixtures (Barlow et al., 2007; Trogisch et al., 2016). Only Vivanco and Austin (2008) designed an experimental setup to isolate the effect of single tree species on litter decomposition. Their study, conducted in Patagonia, found that decomposition in situ is significantly higher after 268 and 366 days, but not before. Therefore, their study supported the HFA hypothesis after a specific time period. However, it is difficult to compare the forest ecosystem in Patagonia to the high diversity of the subtropical forest in our study. Our methodological approach here clearly outlines that it is important to test the HFA hypothesis at small spatial scales and in diverse forests. In addition, this is one of only a few studies conducted in a subtropical forest (but see Liu et al., 2005; Trogisch et al., 2016; Table 1).

In terms of total litter decomposition, litter mass loss was higher here than other studies. For instance, in the study by Chomel et al. (2015) conducted in a boreal ecosystem, litter mass loss ranged between 40% and 53% after two years, less than the overall 78% of mass loss found here. A study from Hobbie et al. (2006) carried out in a maritime–continental climate measured a mass loss of 29–48% after two years, again a considerably lower decomposition compared to our study. These differences are expected, as climate is known to play a major role on litter decomposition at the global level (Meentemeyer, 1978; Aerts, 1997; Keiser and Bradford, 2017), with slower decomposition in colder climates (Liu et al., 2005; Bradford et al., 2016; Rubenstein et al., 2017). Our results are closer to what was described by McGuire et al. (2010) in a tropical forest, where they reported a mean percentage mass loss of 81% in a mixed forest after two years. Liu et al. (2000) assessed litter decomposition of three canopy tree species in Ailao Mountain, the same forest site used here, and they also used two of the same species: *C. wattii* and *L. chintungensis*. Their study was terminated after 22 months, two months earlier than the study presented here, but found similar but lower values of litter mass loss: 75.8% vs. 87.5% for *C. wattii* and 65.6% vs. 71.1% for *L. chintungensis*. It is reasonable to assume that the decomposition measured two months longer will be higher when compared to Liu et al. (2000), as litter decomposition rates decrease exponentially over time (Couteaux et al., 1995; McGuire et al., 2010). This might also be due to changes in the climatic conditions, specifically temperature, which has increased in the past 40 years in the area (He and Zhang, 2005) and is known to influence decomposition (Rubenstein et al., 2017). Alternatively, these results may simply be due to unpredictable stochastic factors.

Soil characteristics varied significantly under different tree species (Appendix 5), as predicted by our second hypothesis. Soil properties are known to present high heterogeneity and to differ both at large and small spatial scales (Waring et al., 2015). Since the study by Zinke (1962) was first published, a considerable amount of literature has provided evidence on the influence of tree species on soil dynamics from all ecosystems: temperate and boreal (Vesterdal et al., 2008), Mediterranean (Aponte et al., 2012), tropical (Russell et al., 2010), desert (Schlesinger et al., 1996), and savanna–grassland (Perakis and Kellogg, 2007). However, these assessments are often conducted in monocultures of single species (Vesterdal et al., 2008; Russell et al., 2010). Analyses on soil heterogeneity in species‐rich forests confirmed the high diversity in soil nutrients even at small spatial scales (Xia et al., 2016), but those results were not linked to the identity of individual tree species. Whether species abundance and/or species diversity shapes soil chemistry (Waring et al., 2015) or whether soil nutrients shape plant communities (John et al., 2007) is still debated.

Here, we show how even within the same forest site, soil properties can change and nutrient relationships can present opposite slopes when sampled under different tree species. For example, the relationship between soil P and soil C presented a negative slope in soil sampled under *M. insignis* and *S. noronhae*, while the slope was positive in soil under *L. chintungensis* and *C. wattii* (Fig. 1). These relationships have been shown to have a positive slope across different ecosystems (Cleveland and Liptzin, 2007), but here we demonstrate how individual tree species can exhibit opposed slopes under their canopy. For the relationship between C and N, the correlation was positive in all cases, and the correlation coefficient was 0.89 overall, which is higher than what was described by Cleveland and Liptzin (2007) in their global review (*R* ^2^ = 0.75). Other soil characteristics, such as pH, total C, total N, available P, and available K, were significantly different among tree species. Soil pH also varied greatly under different tree species in a common garden experiment conducted in Poland (Reich et al., 2005) and in monospecific stands in a deciduous forest in Germany (Schmidt et al., 2015). In the latter, total C also varied between different stands, but total N and total P did not. Moreover, Dawud et al. (2016) described the major role of tree identity as a main driver of soil pH values. Our results support these findings, indicating that this also occurs in species‐rich forests. However, the soil characteristics described in Vivanco and Austin (2008), where the soil samples were collected under “triangles” (three neighbor individuals) of the same species, were similar among microsites, which differs from the results presented here. We are not certain as to the cause and effect or directionality of this relationship, i.e., whether the tree species identity affects the soil nutrients or whether the soil nutrients determine the species distribution (John et al., 2007; Waring et al., 2015). Because of the peculiarity of soil nutrients encountered under the canopy of different species in our study, the ratio between soil C, N, and P varied. The contrasting relationships between soil C, N, and P observed under each tree species could be simply explained by the differences observed in the soil nutrient conditions under each canopy. Furthermore, the differences in litter nutrient content among species may also contribute to the patterns observed here. Further investigations on the relationships between tree species and the soil beneath their canopies are needed to better disentangle the directions of these interactions and their causes.

Soil microbial C and N biomass, which were measured as a proxy for soil microbial biomass, differed among tree species, as suggested by our third hypothesis. The strong correlation between microbial C and N was expected (Cleveland and Liptzin, 2007). However, this correlation varied widely among species, even though it was always positive and significant, with *L. chintungensis* in particular having a smaller slope coefficient. Likewise, because of the strong correlation between C and N, microbial biomass C and N were both higher in *L. chintungensis* and lower in *C. wattii*. These results are in accordance with Bauhus et al. (1998), who showed that tree species composition affected the forest floor microbial biomass. The hypothesis that differences in microbial biomass are influenced by litter chemistry has been supported by a laboratory experiment conducted by Fanin et al. (2014), and this may be a valid explanation for our field study. We acknowledge the fact that microbial biomass P was not estimated, and that the information on microbial biomass presented here is incomplete. Nonetheless, we provide some evidence of the role of tree species identity on forest floor microbial dynamics. More in‐depth studies on microbial biomass and community composition will improve our understanding of the processes that influence microbial biomass.

In conclusion, we demonstrated how forest floor dynamics, here measured as litter decomposition, soil nutrients, and microbial biomass, are linked to tree species identity. Although similar findings were reported in previous studies, our study expands this body of knowledge to an understudied ecosystem and to a highly diverse forest. The methodological approach of our study, using a fine‐scale and species‐by‐species analysis of litter decomposition, together with soil nutrient concentrations and microbial biomass, provided a more nuanced view of forest floor functions. We recommend this approach, especially when dealing with species‐rich forests.

## AUTHOR CONTRIBUTIONS

S.X. and X.Y. designed the study and conducted the field and laboratory work; F.M., S.X., and U.M.G. conducted the statistical analysis; F.M. and U.M.G led the writing; and all authors revised the manuscript and critically contributed to the final version.

## Supporting information


**APPENDIX S1.** Percentage of litter mass loss under different tree species. Species on the *x*‐axis represent litter species. (A) Percentage of litter mass loss under *Castanopsis wattii*; (B) percentage of litter mass loss under *Lithocarpus chintungensis*; (C) percentage of litter mass loss under *Manglietia insignis*; (D) percentage of litter mass loss under *Schima noronhae*.

**APPENDIX S2.** Differences in microbial biomass of nitrogen (A) and carbon (B) under different tree species. Significant differences are shown with different letters. Species names on the *x*‐axis represent the tree species under which the soil was sampled.

## APPENDIX 2 Description of the four selected species used in this study.^a^

**Table:**

Species	Family	Mature height	Occurrence in forest strata	Shade tolerance	Geographic distribution
*Castanopsis wattii* A. Camus	Fagaceae	15–20 m	Canopy	Shade tolerant	China (Tibet, Yunnan), India (Sikkim, Assam)
*Lithocarpus chintungensis* Hsu & Qian	Fagaceae	<15 m	Canopy	Shade tolerant	China (south‐central, southeast), Taiwan
*Manglietia insignis* Blume	Magnoliaceae	30 m	Canopy	Shade tolerant	China (southern), India (Assam), Myanmar, Nepal, Thailand, Vietnam
*Schima noronhae* Reinw.	Theaceae	20 m	Canopy	Intermediate light demand	China (Yunnan), Indonesia, Laos, Malaysia, Myanmar, Thailand, Vietnam

a Information was obtained from eFloras (2008).
