# Supplementary material for: Small‐scale and multi‐species approaches for assessing litter decomposition and soil dynamics in high‐diversity forests
Source: Appl Plant Sci. 2019 Apr 19;7(4):e01241. doi: 10.1002/aps3.1241 (PMC6476167; doi:10.1002/aps3.1241)

Martini et al.—Applications in Plant Sciences 2019 7(4)—Data Supplement S1
DOI

APPENDIX S1. Percentage of litter mass loss under different tree species. Species on the *x*-axis represent litter species. (A) Percentage of litter mass loss under *Castanopsis wattii*; (B) percentage of litter mass loss under *Lithocarpus chintungensis*; (C) Percentage of litter mass loss under *Manglietia insignis*; (D) percentage of litter mass loss under *Schima noronhae*. Gray box plots represent the same species of litter and tree. Different letters represent significantly different percentage of litter mass loss between the litter from the same tree species (gray box plot) and each of the other litter species (white box plots), but not between the white box plots.

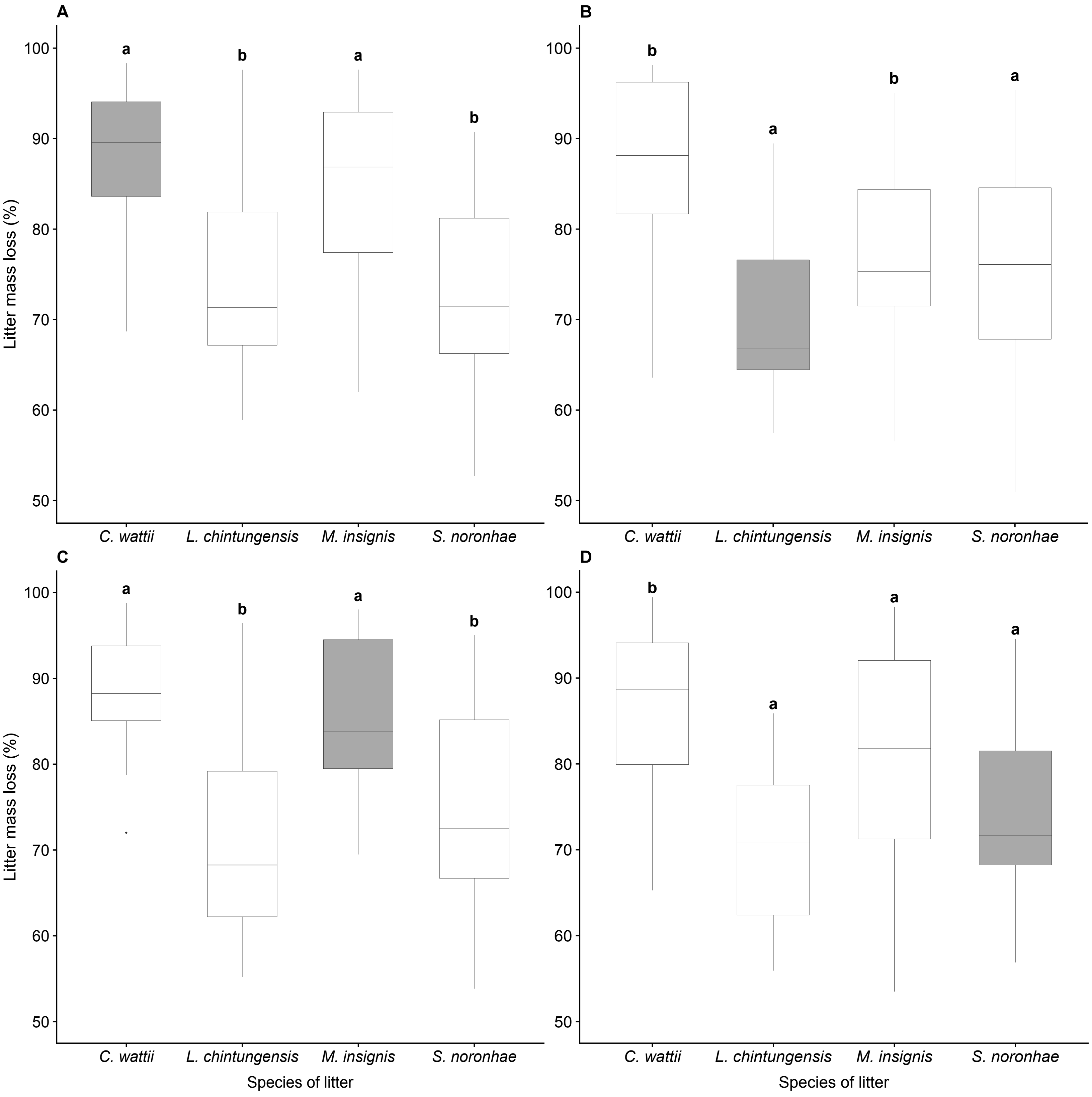

Supplement: Supplementary file 1 — APPENDIX S1. Percentage of litter mass loss under different tree species. Species on the x‐axis represent litter species. (A) Percentage of litter mass loss under Castanopsis wattii; (B) percentage of litter mass loss under Lithocarpus chintungensis; (C) percentage of litter mass loss under Manglietia insignis; (D) percentage of litter mass loss under Schima noronhae. [file APS3-7-e01241-s001.pdf]
