# Supplementary material for: Small‐scale and multi‐species approaches for assessing litter decomposition and soil dynamics in high‐diversity forests
Source: Appl Plant Sci. 2019 Apr 19;7(4):e01241. doi: 10.1002/aps3.1241 (PMC6476167; doi:10.1002/aps3.1241)

Martini et al.—Applications in Plant Sciences 2019 7(4)—Data Supplement S2
DOI

APPENDIX S2. Differences in microbial biomass of nitrogen (A) and carbon (B) under different tree species. Significant differences are shown with different letters. Species names on the *x*-axis represent the tree species under which the soil was sampled.

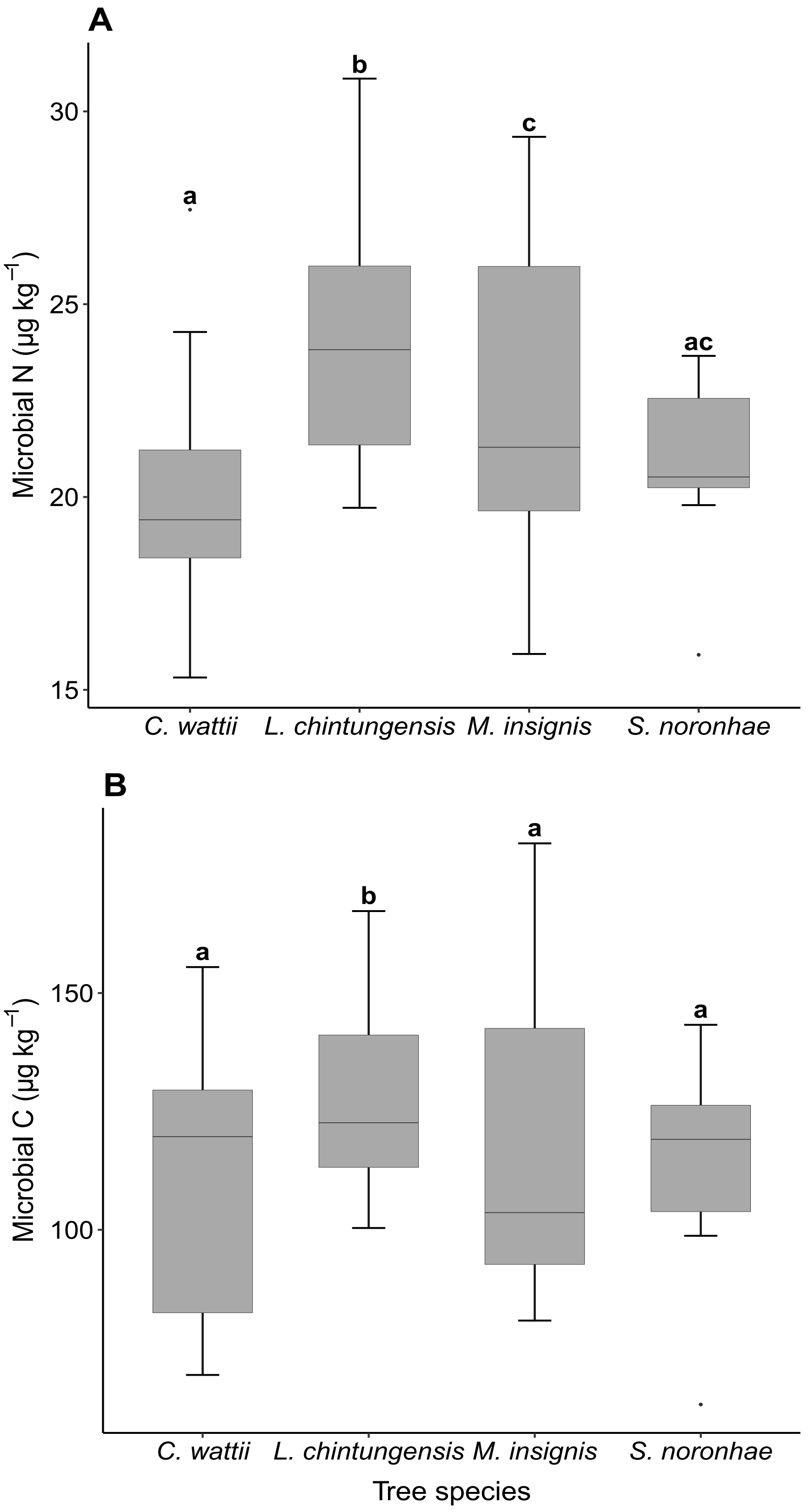

Supplement: Supplementary file 2 — APPENDIX S2. Differences in microbial biomass of nitrogen (A) and carbon (B) under different tree species. Significant differences are shown with different letters. Species names on the x‐axis represent the tree species under which the soil was sampled. [file APS3-7-e01241-s002.pdf]
